# Neural Tissue Degeneration in Rosenthal’s Canal and Its Impact on Electrical Stimulation of the Auditory Nerve by Cochlear Implants: An Image-Based Modeling Study

**DOI:** 10.3390/ijms21228511

**Published:** 2020-11-12

**Authors:** Kiran Kumar Sriperumbudur, Revathi Appali, Anthony W. Gummer, Ursula van Rienen

**Affiliations:** 1 Institute of General Electrical Engineering, University of Rostock, 18051 Rostock, Germany; revathi.appali@uni-rostock.de (R.A.); ursula.van-rienen@uni-rostock.de (U.v.R.); 2 Ageing of Individuals and Society, Interdisciplinary Faculty, University of Rostock, 18051 Rostock, Germany; 3 Department of Otolaryngology, University of Tübingen, 72076 Tubingen, Germany; anthony.gummer@uni-tuebingen.de; 4 Life, Light and Matter, Interdisciplinary Faculty, University of Rostock, 18051 Rostock, Germany

**Keywords:** neurodegeneration, tissue density, auditory nerve, cochlear implant, modeling and simulation, finite element models, image-based modeling

## Abstract

Sensorineural deafness is caused by the loss of peripheral neural input to the auditory nerve, which may result from peripheral neural degeneration and/or a loss of inner hair cells. Provided spiral ganglion cells and their central processes are patent, cochlear implants can be used to electrically stimulate the auditory nerve to facilitate hearing in the deaf or severely hard-of-hearing. Neural degeneration is a crucial impediment to the functional success of a cochlear implant. The present, first-of-its-kind two-dimensional finite-element model investigates how the depletion of neural tissues might alter the electrically induced transmembrane potential of spiral ganglion neurons. The study suggests that even as little as 10% of neural tissue degeneration could lead to a disproportionate change in the stimulation profile of the auditory nerve. This result implies that apart from encapsulation layer formation around the cochlear implant electrode, tissue degeneration could also be an essential reason for the apparent inconsistencies in the functionality of cochlear implants.

## 1. Introduction

An estimated 466 million people, including 34 million children, are subjected to disabling hearing loss worldwide [1]. A variety of physical, genetic, and pathological factors cause different degrees of hearing loss [2,3,4]. Sensory neural deafness is a consequence of a loss of peripheral neural input to the auditory nerve (AN), which can be due to neural degeneration in the presence of a normal compliment of cochlear hair cells, called “primary” neural degeneration, or due to the loss of inner hair cells followed by causal neural degeneration, called “secondary” neural degeneration [5]. Of course, a loss of neural input can also be due to the malfunction of cochlear components, without necessarily implying anatomical absence. In the absence of peripheral neural activity, the spiral ganglion neurons (SGNs) when intact with the central processes in Rosenthal’s canal (RC), provide a window of opportunity for clinical intervention with a cochlear implant (CI) [6,7]. The CI consists of an array of electrodes inserted into scala tympani to stimulate SGNs electrically [8,9]. The electrical stimulation of the AN by CIs facilitates hearing in the deaf or severely hard-of-hearing [10,11,12].

**Figure 1:**
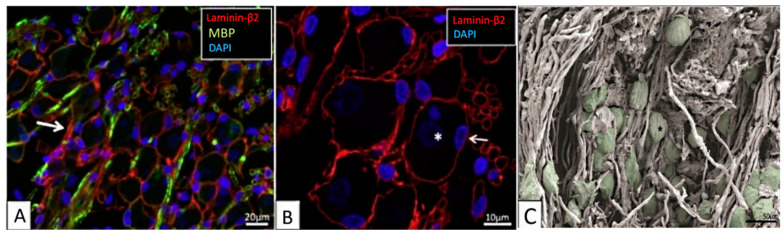
The human spiral ganglion. (**A**,**B**) Confocal-microscopic images showing immunoreactivity of laminin-β2, myelin basic protein (MBP), and 4′,6-diamidino-2-phenylindole (DAPI) stained cell nuclei. (**A**) Most type-1 spiral ganglion neurons (SGNs) are MBP-negative. Some non-myelinated perisomal segments show a rich expression of laminin (arrow). (**B**) Laminin-β2 immunoreactivity of basement membrane lining the extracellular surface of the satellite glial cells (SGCs) of the SGN bodies and nerve fibers. Type-1 SGN cell nuclei are round and darkly stained (*), while SGC nuclei are often crescent-like (arrow) and more lucent. Their cytoplasm shows no laminin expression. (**C**) Scanning-electron-microscopic image of the human spiral ganglion. Neural cell bodies are artificially colored green. The cell coat of several neurons shows longitudinal impressions (*) caused by crossing myelinated nerve fibers. Images are from the basal cochlear turn from subjects with normal pure-tone thresholds for their age (40–65 years). Adapted from [16], Figures 2A,B and 6A with permission.

Various experimental studies and mathematical models have attempted to explain how the electric field produced by the CI stimulates the AN in RC [13]. In particular, mathematical models have been harvesting rich experimental data and serving as useful tools for investigating the influence of different electrophysiological parameters on the efficacy of the CI. However, mathematical models of a bioelectric phenomenon rely upon several model assumptions and geometrical simplifications [13,14,15], which can result in impractical modeling outcomes. For example, histological studies [16,17,18,19] demonstrate that RC is filled with various neural tissues such as type-1 and type-2 SGNs, satellite glial cells, peripheral and central processes of the AN; examples are shown in Figure 1. In spite of this rich tissue heterogeneity, the state-of-the-art mathematical models have assumed a homogeneous extracellular medium to model the electric-field distribution in RC. With such an assumption, the existing in-silico models have shown that the electrode placement in scala tympani [20,21], cochlear anatomy [22,23], electrode and stimulating pulse configurations [24,25,26,27], and the dielectric properties of cochlear tissues [28,29,30] are some of the important factors to be considered for optimal stimulation of the AN by the CI. A few in-silico studies have incorporated the neural tissues, particularly AN processes, in the modeling [31,32,33], but they have not considered the distribution of heterogeneous tissues in RC. Apart from those reports, in-silico studies have not explicitly investigated the effect of neural tissue depletion on AN stimulation by the CI. The present simulation study investigates the electrical effect of neural tissue loss on the CI responses of the AN.

In contrast to the existing in-silico models, in our earlier computational study [34], we modeled a heterogeneous medium composed of different neural tissues in RC and studied the effect of neural tissue heterogeneity on the electrical stimulation of type-1 SGNs. The external electric field induces a cell-specific potential difference across the high-resistive cell membrane. The difference between the extracellular and intracellular potentials is defined as the transmembrane potential (V_m_). When V_m_ crosses a threshold potential, neural excitation takes place in the form of an action potential (AP) [35]. Below this threshold, the membrane behaves approximately as a linear system containing only resistance and capacitance [36], so that the sub-threshold response of a membrane complex can be evaluated by superposition of the component membrane responses [37]. The probable initiation site of an AP can be estimated on the basis of V_m_ relative to the threshold potential [37]. By comparing the maximum value of V_m_ relative to an ad hoc threshold, we have shown, theoretically, that the neural tissues around the SGNs alter the electric-field distribution in RC and thereby influence the excitation profile of the AN. It was shown that the orientations of the SGNs relative to the applied electric field also play a vital role in AP initiation and, thereby, stimulation of the AN. Furthermore, we showed that if many passive neural tissues encompass the SGNs, then the number of surviving SGNs has little impact on AP initiation. Finally, the electrode distance from RC appeared to only mildly affect AN stimulation.

In the present paper, we investigate how the change in neural tissue density in RC may alter the putative AP initiation sites. Numerous histological studies of the cochlea have reported neural degeneration with age [38,39,40], whereby the degeneration can be either primary or secondary [5,38,41]. A post-mortem study of human tissue has estimated that for each 10% of total neuronal loss, primary neural degeneration causes average thresholds at each audiometric frequency to increase by 6 dB hearing level and a word recognition score to decrease by 6.8% [38]. Although there are a multitude of factors influencing the clinical success of a CI, the number of surviving SGNs and central processes, the presence or absence of peripheral dendrites, as well as the relative duration of hearing before the onset of hearing loss are crucial [42,43,44]. Intuitively, these observations prompt further investigation to decide whether CI functionality depends on the tissue density in RC. Such an investigation is essential, since patients with profound deafness, especially children, can carry the CI their entire life, in which time progressive neural degeneration as well as the loss or production of various fibrous tissues in RC is possible [41,45,46,47,48]. Changes in the densities of neural and aneural tissues in the RC might affect the performance of the CI in an unpredictable manner. In this context, theoretically studying the response of SGNs to the applied electric field is a logical starting point, since AP generation arguably takes place on the axonal initial segments or cell bodies of the SGNs in RC [49,50,51,52].

Several dielectric tissue interfaces, such as modiolus bone, tympanic fluid, and myelinated neural tissues and other cells are situated between the CI electrode and the SGNs. In the modeling perspective, the geometry and dielectric properties of each tissue in the region of interest are essential for a realistic description of the associated bioelectric phenomena. Due to insufficient morphological and biophysical data, as well as modeling limitations, it may not be possible to accurately implement the topology of all tissues and their dielectric properties in a modeling and simulation study. For example, in the present case for RC, the electric conductivity values of tissues such as central processes of the AN, satellite glial cells, type-2 SGNs, other myelin tissues, Schwann cells, and the bony labyrinth are, to a large extent, unknown. In addition, large variations in the shape and size of the human cochlea have been reported [53,54,55]. Therefore, at present, even a realistic model of the cochlea would not be sufficient to study patient-specific simulation scenarios, which is a goal of personalized medicine.

The main focus of the present study is modeling heterogeneous tissues of irregular shapes and distribution patterns in RC and then determining their effects on the electric fields and transmembrane potentials. To date, there is no template geometry available for modeling the neural tissues in RC. We have addressed this issue with the aid of an immunohistochemical image of RC containing all the necessary neural tissues. Instead of modeling the geometry of randomly distributed tissues as a subdomain, we use our image-based method [34] to define the dielectric properties of the neural tissues from color-coded immunohistochemical images of the RC. Otherwise, modeling the heterogeneous tissue geometry in RC by image segmentation methods [56] would be not only laborious but also implausible due to the meshing complexities demanding high computational expense in finite-element analysis. Moreover, the implementation of gradual tissue depletion would not be possible if the geometry of the tissues was fixed by the segmented model.

Here, we show that a loss of neural tissue in the vicinity of SGNs can cause disproportionate changes of the electric fields and transmembrane potentials in a manner that is not predictable from the size of the SGNs and their distances from the stimulus electrode.

## 2. Results

We present the results of an in-silico simulation study of the responses of SGNs in Rosenthal’s canal to electrical stimulation with a cochlear implant in scala tympani. The model is based on a finite-element model of the electrical conductivities of neural and aneural tissues identified with image analysis of an immunohistochemically stained material. The degeneration of all neural tissue except SGNs and satellite glial cells was emulated by changing the pixel intensity. Responses to voltage steps are illustrated for the maximum transmembrane potentials (Section 2.1) and for the electric fields (Section 2.2), for both normal and degenerated neural tissue.

### 2.1. Dependence of the Maximal Transmembrane Potential on Tissue Density

**Figure 2:**
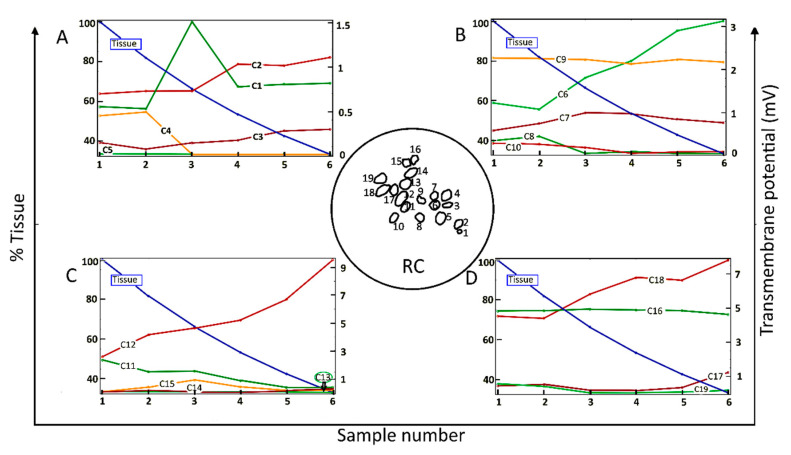
Maximum values of the transmembrane potential, V_m_, elicited by an applied electric field for spiral ganglion neurons in a two-dimensional section of Rosenthal’s canal (RC); see the circular inset, as a function of the relative amount of surrounding neural tissue. Maximal V_m_ is displayed for six relative values (Sample number) of the amount of tissue (% Tissue). There are 19 cells in the computational section of RC, denoted by C1–C19; the data are distributed across four panels (**A**–**D**). In this and the following figures, the stimulus voltage is 1 V. Taken as a group, there was no correlation of maximal V_m_ with the amount of tissue.

Figure 2 shows the dependence of the maximum value of V_m_ for each of 19 neighboring cells (C1–C19) in a two-dimensional section of RC (inset of Figure 2) as a function of the amount of surrounding neural tissue. Tissue reduction was achieved by systematically introducing a parameter α into the conditional equation describing the electrical tissue conductivity as a function of pixel intensity (Section 4, Equation (1)). Maximal V_m_ values are shown for six values (“samples”) of the relative amount of tissue.

Taken across the population of 19 cells, there was no global dependence of maximal V_m_ on the amount of tissue. For example, for the original amount of tissue, denoted by Sample-1 and taken directly from the immunohistochemical image from a human subject, the maximal induced V_m_ on C1 is 0.5 mV, and if the neural tissue is decreased to 80%, as in Sample-2, the maximal V_m_ on C1 does not change significantly. However, if the tissue density is further decreased to 65%, as in Sample-3, the maximal V_m_ for C1 increases to 1.5 mV. In the remaining samples, the maximal V_m_ on C1 is constant at 0.8 mV. The responses of C9 and C16 do not depend on the amount of neural tissue. In contrast, the maximal V_m_ at C2, C3, C6, C12, and C18 increase approximately monotonically with the loss of surrounding neural tissue. Other cells tend to produce smaller V_m_ with increasing tissue depletion (C4, C5, C8, C10, C11, and C19).

Taken together, the simulation results presented in Figure 2 imply that SGNs and their total number producing electrically evoked APs depend on the amount of surrounding tissue, but not necessarily in a predictable manner. Taking the maximum value of V_m_ as an indicator of the likelihood of AP generation and if the threshold potential for eliciting an AP were, say, 1 mV, then for the largest amount of tissue (Sample-1), six of the 19 SGNs would yield APs (C6, C9, C11, C12, C16, and C18). The same six cells would produce APs for tissue reduction to 80% (Sample-2). However, for tissue reduction to 65% (Sample-3), a further two cells would yield APs (C1 and C7), and for tissue reduction to 40% (Sample-5), another group of six cells would produce APs (C2, C6, C9, C12, C16, and C18). Clearly, the cells and their proportion in RC producing APs not only depend on the surrounding neural tissue but also on the value of the threshold potential.

### 2.2. Dependence of the Electric Field on Tissue Density

**Figure 3:**
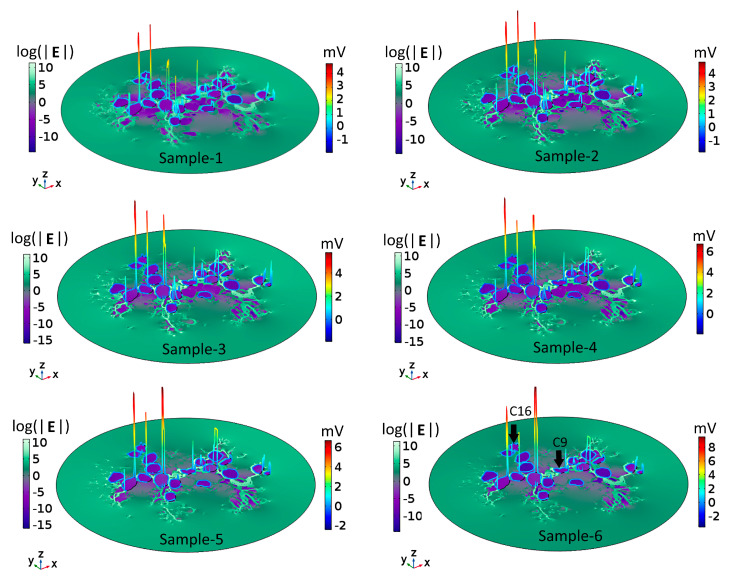
Electric field, **E**, and maximal transmembrane potential, V_m_, for the six tissue samples shown in Figure 2. The color-coded peaks (blue-to-red) show the sites of maximal induced V_m_ on the spiral ganglion neurons. The distribution of **E** in Rosenthal’s canal is shown with a logarithmic scale (log(|**E**|) for better visualization; the reference field amplitude is 9 × 10^4^ V/m. The Cartesian coordinate axes denote the image plane (x,y) and the dependent variable (z); namely, log(|**E**|) or the maximum of V_m_. The formation of electric field “bright spots” (blue-to-white peaks) in the vicinity of neural tissues is due to the heterogeneous conductivity distribution of respective neural tissues. The black arrows in Sample-6 point to C9 and C16: their V_m_ remained the same across samples because the density of GroupB tissues was not changed in any of the samples.

To understand the voltage profiles presented in Figure 2, Figure 3 displays the electric-field distribution for each of the tissue samples. Since neural tissues in RC have lower conductivity than that of the extracellular medium, “bright spots” (blue-white peaks) of the electric field can be seen in the vicinity of neural tissues of very low electric conductivity. The presence of these peaks shows that neural tissue alters the electric field distribution around the SGNs, which also then affects the V_m_. In all six samples, the density of GroupB tissue, defined as all tissue except satellite glial cells and SGNs (see Section 4), around C9 and C16 has not changed; hence, the V_m_ of those two SGNs remains unaltered. Color-coded peaks (blue-to-red) in Figure 3 show the sites of maximal induced V_m_ on the SGNs.

**Figure 4:**
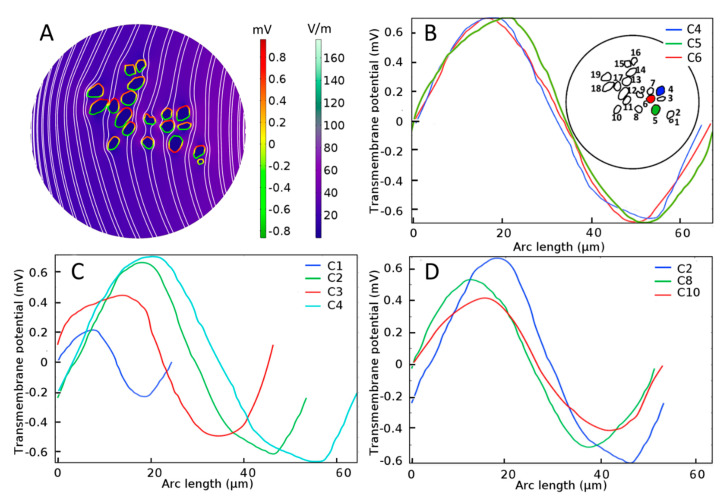
Electrical stimulation of spiral ganglion neurons (SGNs) in a homogeneous extracellular medium. (**A**) Color-coded representation of electric-field distribution in Rosenthal’s canal. The formation of hyperpolarization and depolarization sites on SGNs can be seen with the red-to-green color code, respectively. Streamlines of the electric field are shown in white. Evoked transmembrane potential, V_m_, on specified SGNs having (**B**) similar sizes and distances from the electrode, where C4, C5, and C6 are shown in blue, green, and red color code, respectively in the inset, (**C**) different sizes but similar distances from the electrode, and (**D**) similar sizes but different distances from the electrode. *Arc length* denotes the perimeter of SGNs.

When a cell that has a uniform membrane is exposed to a uniform electric field, hyperpolarization and depolarization occur at diametrically opposite sites, as shown in Figure 4A. However, in the present case of nonhomogeneous tissue, the cell membrane of each SGN is enveloped by GroupA (satellite glial cells; see Section 4) and GroupB tissues, which act as additional non-uniform membrane patches. Hence, the effective electric conductivity of the membrane varies locally around each SGN, which in turn affects the intracellular fields [57]. In such a scenario, the locations of maximal and minimal induced V_m_ on a cell depend upon where the current enters and exits the cell [58]. The current entering the cell through a high electrical conductive patch must pass out through the remaining low conductive patch to maintain the continuity of the current flow. This implies that the distribution of tissue patches around the cell membrane determines the response of SGNs to the applied electric field. As a consequence, even a small change in the tissue density in RC could impact on the tissue patch distribution around one or the other SGN and, thereby, on its excitation.

## 3. Discussion

We have investigated the effect of neural tissue in Rosenthal’s canal (RC) on the transmembrane potential (V_m_) and the initiation sites of action potentials (APs) in response to electric fields produced by intracochlear stimulation from a cochlear implant (CI). Tissue heterogeneity in RC was implemented with a simple yet effective image-based method, which otherwise would have been an implausible task with conventional modeling. Based on the imaging method, we have described the heterogeneity of the passive electrical conductances of the tissues in RC and, thereby, within the framework of a finite-element analysis, been able to solve the electrical constitutive equations to derive the electric fields, current densities, and voltages. The model shows that the spatial distribution of the V_m_ critically depends on the amount of tissue. An immediate prospect of the present study would be revisiting contextual state-of-the-art in-silico models for the possible implementation of tissue heterogeneity. In the long term, the results should provide an impetus for considering heterogeneous neural tissue properties when optimizing CI stimulus conditions, which is a topic that is addressed in the following discussion. The results can also be applied to the electrical stimulation of other tissues in the body [59,60,61].

### 3.1. Neural Stimulation in Homogeneous and Inhomogeneous Tissue Environments

For SGNs exposed to a uniform external electric field in a homogeneous extracellular medium such as shown in Figure 4A, the induced V_m_ depends on the cell shape and size [62] as well as distance from the stimulating electrode. From Figure 4B, it is observed that the maximal induced V_m_ in the case of a homogeneous medium is similar (0.7 mV) for three SGNs of similar sizes and distances from the electrode (C4, C5, and C6). For similar distances from the electrode, Figure 4C shows that the induced V_m_ is approximately proportional to the cell size (C1, C2, C3, and C4). For similar cell sizes, Figure 4D shows that the induced V_m_ is proportional to their distances from the electrode (C2, C8, and C10).

Based on such results under homogeneous conditions, it would almost certainly be concluded that for a given voltage-stimulus paradigm at some longitudinal position along the cochlea, for example in the basal turn where most type-1 SGNs have a diameter of approximately 30 µm [63], the electrode distance from the SGNs is an essential design factor to be considered for achieving optimal excitation of the AN. Under the homogeneity assumption, any mathematical model would almost certainly infer that an electrode placed nearer to the modiolus in scala tympani, such as a modiolus hugging electrode, would perform better compared to a more laterally placed electrode [50,64,65,66].

**Figure 5:**
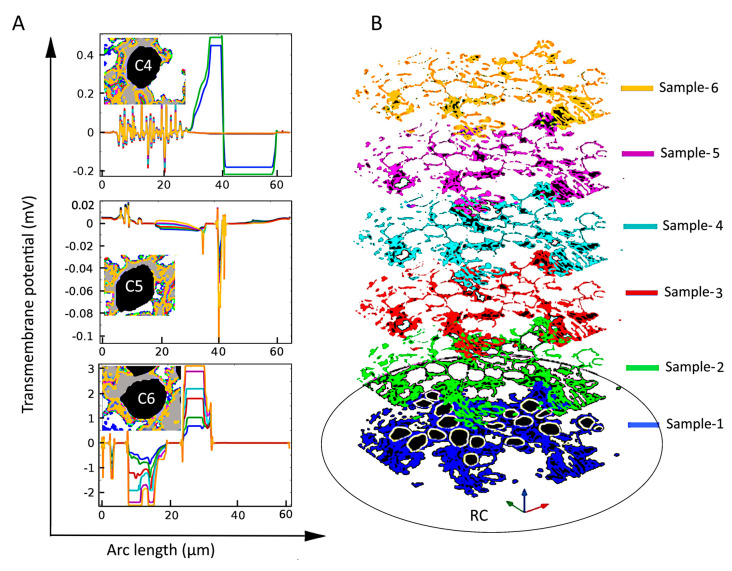
The effect of tissue density (**B**) on the induced transmembrane potential V_m_ (**A**) for spiral ganglion neurons (SGNs: C4, C5, and C6) of similar sizes and distances from the stimulating electrode. Insets in the left panels: Local view of encapsulation tissue in the vicinity of the indicated SGN. Notice the strong and apparently random dependence of V_m_ on the amount of tissue both across cells and for a given cell.

However, under inhomogeneous conditions, the present analysis shows that neighboring SGNs of similar size can produce V_m_ of vastly different amplitude and spatial distribution. Compare, for example, the spatial distributions of V_m_ for the three cells shown in Figure 4B and Figure 5 (C4, C5, and C6) under homogeneous and inhomogeneous conditions, respectively. Referring to the tissue profiles of the six samples in Figure 5, we observe that despite their similar size and location in RC, V_m_ is vastly different across cells for a given tissue condition and for a given cell for different amounts of tissue. It has been shown that the site of AP generation is determined by specific ion-channel distributions on the cell membrane or the axonal initial segments [51,67]. In the present study, the ion channel distribution on the cell membrane was not included, yet the V_m_ exhibited strikingly different spatial properties dependent on the amount and type of neural tissue and, therefore, on the resulting inhomogeneous profile of the electrical conductances. Moreover, referring to Figure 2, we see that although cells C16 and C18 are much further from the electrode than cells C1–C4, their larger values of maximal V_m_ mean that they are more likely to produce APs than the proximally located cells.

Taken together, these results imply that rather than simply the position of the electrode relative to the SGNs, the local tissue density around each SGN is of paramount importance for the initiation site of an AP. In other words, any theoretical correlation between the electrode placement in scala tympani and the excitation of SGNs and, thereby, the quality of speech perception would be speculative. Importantly, such correlation has not been observed in experimental studies [68,69,70].

### 3.2. The Activating Function as an Indicator of AP Initiation Site

**Figure A2:**
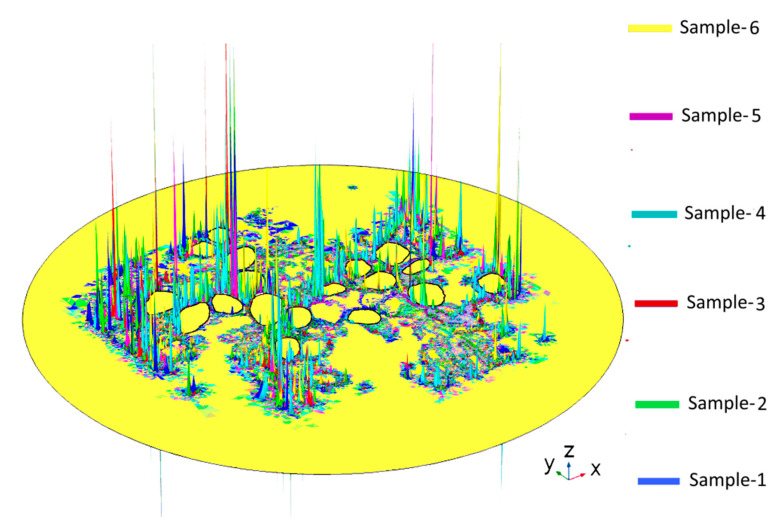
Activating functions in Rosenthal’s canal for the six tissue samples used in the study. The maximum of an activating function is often used as an indicator of the probable AP initiation site on the neural tissue. Here, the locations of the maxima appear to change randomly with change in tissue density.

**Figure A3:**
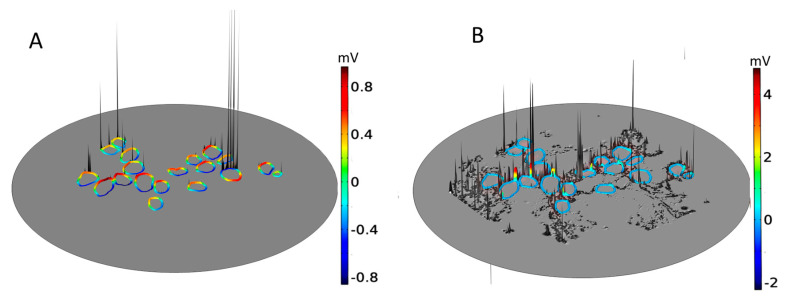
The activating function and transmembrane potential (V_m_) in Rosenthal’s canal for a homogeneous extracellular medium (**A**) and Sample-1 (**B**). The maxima of the activating function shown in gray code and the maxima of V_m_ shown in blue-to-red color code represent two models for predicting the most probable AP initiation sites on the neural tissues. A correlation between the two sets of maxima was not detected.

Most of the mathematical, AP-generating models of the cochlea have used the maximum of the second spatial derivative of the extracellular potential, known as the “activating function” [71], as an indicator of the most likely site of AP initiation [14,20,23,32,72,73,74]. The activating function evaluated for the 19 SGCs and six tissue samples reveals peaks with positions that change with tissue density, in what appears to be an unpredictable manner (Appendix B, Figure A2). This observation is consistent with the strong interdependency of neural excitation and neural density in RC provided by the analysis of the spatial distributions of the V_m_ and the electric field, and their dependence on tissue density (Section 2.2). Importantly, we did not detect a correlation between the peaks of the activating function and those of V_m_ (Appendix B, Figure A3). This result is consistent with those studies that report that the maximum of the activating function does not always accurately predict the AP initiation site [75,76,77,78].

### 3.3. Limitations of the Analysis

Being a first-of-its-kind study, our mathematical model suffers a few drawbacks, such as an unavailability of sufficient image data to reconstruct a realistic three-dimensional model of the RC filled with heterogeneous tissue. However, we carefully chose an immunohistochemical image that contains SGNs of different size, shape, and sufficiently different neural tissues to represent possible variations in RC throughout the cochlea. Thus, the two-dimensional representation of the region of interest is a first approximation of AN stimulation with the CI. Nevertheless, the image-based method proposed for modeling two-dimensional heterogeneous tissues can be readily extended to a three-dimensional model derived from a stack of immunohistochemical images of RC. However, a three-dimensional realistic model with heterogeneous tissues in RC is expected to produce qualitatively similar results. Since, for bipolar stimulation, the electric field would be confined to a small region, the anatomy of the whole cochlea would have little impact on the stimulation profile of the SGNs.

One of the biggest challenges to produce in-silico study results comparable to those expected in vivo is the unavailability of accurate dielectric properties of various cochlear tissues. Moreover, comprehensive information about the types and distributions of the ion channels responsible for the excitation of SGNs is lacking [79]. Prospectively, a recent study attempted to find the threshold currents of SGNs by culturing them on a multi-electrode array system [80]. The response of SGNs to an applied electric field could also vary with their position along the cochlea [81,82]. The threshold potential of SGNs can be estimated with Hodgkin–Huxley theory by taking a limited number of ion channels into account [83]. Nevertheless, since the V_m_ is essential for neural excitation, a systematic study such as the present one does provide initial information about possible AP initiation sites and their dependence on tissue density. Clearly, our in-silico study needs to be validated by appropriate experimental studies. To the best of our knowledge, there are no experimental studies with which our in-silico results can be compared.

### 3.4. Potential Clinical Applications

As discussed in [84], CI research is facing challenges to find the underlying factors responsible for the unusual variability in CI performance across children and adults. The performance of the CI can change with time after implantation due to the formation of an unwanted tissue layer, or so-called encapsulation layer, around the stimulating electrode [85]. It is also possible that unwanted tissue develops on the basilar membrane (far) apical from the distal end of the CI electrodes [86]. Therefore, extending the results of the present study to the whole length of the cochlea, we suggest that even a small change in tissue density in RC could disproportionately affect electrical stimulation of the AN along the entire cochlea. Hence, reported inconsistencies in the performance of CIs [87,88,89] could be partially attributed to the eventual degeneration of (heterogeneous) neural tissues in RC and perhaps even the generation of aneural tissue. A few in-silico studies have proposed patient-specific and fully automated models to aid CI surgery and evaluation [90,91]. However, a reliable method or model that can be clinically used as a template or a pre-implant predictor for CI functionality is not yet available. Anticipating as many influencing factors as possible is obviously essential for such an endeavor. In this sense, the results of the present study propose tissue density in RC as an influencing factor that might affect CI performance and, therefore, it needs to be considered when attempting to optimize the design and performance of the CI. The results might also serve as an initial framework for modeling the latest CI developments such as the drug-induced artificial regrowth of neural tissues in RC [79,92,93,94,95].

## 4. Materials and Methods

Here, we briefly present the image-based modeling of the electrical conductivities and CI-induced electric fields for heterogeneous neural tissues in RC; refer to our earlier work [34] for a comprehensive description of the method. In addition, we describe a novel means of altering tissue density in RC to simulate degeneration of tissue surrounding SGNs. All modeling and simulation tasks were performed with the AC/DC module in a commercially available FEM software package (COMSOL Multiphysics^®^, Simulation software version 5.3a; Stockholm, Sweden).

### 4.1. Basic Assumptions

**Figure 6:**
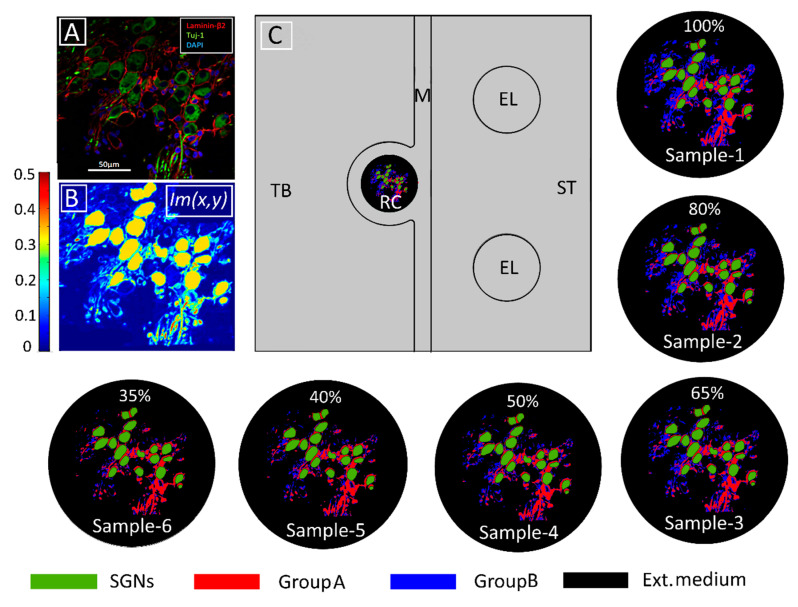
Modeling tissue density and its change. (**A**) Immunohistochemical image of human Rosenthal’s canal in which spiral ganglion neurons (SGNs), satellite glial cells, and cell nuclei are marked with Tuj-1 (green), Laminin-β2 (red), and DAPI (blue), respectively. Extracellular medium is indicated in black. (From [16] Figure 2E with permission) (**B**) Pixel intensities represented by the Im(x,y) function from COMSOL Multiphysics^®^5.3a. (**C**) Model geometry in the computational domain consisting of temporal bone (TB), Rosenthal’s canal (RC), modiolus (M), electrodes (EL), and scala tympani (ST). The conductivity distribution of the original tissue (Sample-1) was calculated according to Equation (1) with α=0 The following five samples represent increasing tissue loss introduced computationally by increasing α in Equation (1) from 0.01 to 0.05 in steps of 0.01, respectively. The loss was introduced into GroupB tissue (all neural tissue except satellite glial cells and SGNs). The percentages represent the amount of GroupB tissue relative to that in the original sample. Panels (**B**,**C**) adapted from [34] with permission.

A two-dimensional finite-element model of the region of interest in the basal region of the human cochlea was used to implement the spatial dependence of the tissue conductivities derived from the immunohistochemical data. The immunohistochemical image is shown in Figure 6A. Given the attempts to focus electric fields in CI design [24,96,97], bipolar as opposed to monopolar stimulation is presumed to be appropriate for examining the effects of localized electric fields on V_m_. The dimensions of the computational subdomains are given in Table 1.

Membranes of all neural tissues are assumed to be passive and resistive because AP generation or propagation in neural tissues was not subsumed in the simulation. This assumption also obviates the need for the physical presence of heterogeneous tissue boundaries in the model. Thus, rather than the actual geometry, the electric conductivity distribution of respective tissues in RC is sufficient to derive the spatial dependence of the electric field and V_m_.

In the model, the neural tissues in RC are classified into two groups: (1) The satellite glial cells, which encapsulate the SGNs, called here GroupA tissues, and (2) all other neural tissues except for SGNs, which are called GroupB tissues. The death of satellite glial cells would trigger the degeneration of SGNs [98]; hence, the loss of GroupA tissues is not modeled. The electric conductivity of GroupA tissues is assumed to be lower than that of the cell membrane [99]. All tissues in GroupB that comprise the central extensions of type-2 SGNs and other neighboring cell bodies are considered to be myelinated tissues.

### 4.2. Derivation of the Distribution of Electrical Conductivities

To model the degeneration of GroupB tissues, the immunohistochemical image of the tissues of interest was delineated with false color code [Figure 6B] and imported into COMSOL Multiphysics^®^ 5.3a. The pixel dimensions of the image were scaled in the model according to the dimensions of the RC subdomain. In this manner, the pixel intensity data of each delineated tissue were allocated according to their scaled spatial coordinates, which were denoted by Im(x,y). Tissue conductivity values (σ_Tissue_) were mapped in the computational domain using the conditional equation:

(1) σTissue={σIntracell,     0.3<Im(x,y)≤0.5σGroupA,    0.15<Im(x,y)≤0.3σGroupB,(0.05+α)<Im(x,y)≤0.15σExtracell,    Im(x,y)≤(0.05+α)

where σ_Intracell_, σ_GroupA_, σ_GroupB_, and σ_Extracell_ are the intracellular, GroupA, GroupB, and extracellular conductivities, respectively. Six samples—namely, Sample-1, Sample-2, Sample-3, Sample-4, Sample-5, and Sample-6—of RC were modeled by assigning 0, 0.01, 0.02, 0.03, 0.04, and 0.05 respectively, to the intensity parameter α. The resulting six tissue samples are presented in Figure 6. To quantify tissue loss, the amount of GroupB tissue present in Sample-1 (the original sample from the immunohistochemical image) was defined as 100% (α = 0), and for every other assigned value of α, the resultant GroupB tissue percentage was calculated relative to the amount of GroupB tissue in Sample-1; these percentages are those given in the respective panels of RC in Figure 6.

Nineteen SGNs were modeled as computational subdomains by extracting the contours of cell bodies from the immunohistochemical image in Figure 6A. Figure 6B depicts the pixel intensities Im(x,y) of the imported image, which is scaled from 0 to 0.5. Table 2 lists the electric conductivity values used for the various types of cochlear tissue; they are adapted from [34]. Using Equation (1), Figure 6 shows the resultant conductivity distribution in RC for all samples.

### 4.3. The Constitutive Equations

The contact impedance boundary condition

(2) $$\begin{array}{c} n\cdot J_{\mathrm{int}}=\sigma_{m}\left(V_{\mathrm{int}}-V_{\mathrm{ext}}\right)/d\\ n\cdot J_{\mathrm{ext}}=\sigma_{m}\left(V_{\mathrm{ext}}-V_{\mathrm{int}}\right)/d\;\\ V_{m}=V_{\mathrm{int}}-V_{\mathrm{ext}}\;\;\;\;\;\;\;\; \end{array}\}$$

was used to electrically describe the thin membrane (d=10 nm) of the SGNs. Here, **n**, **J**, $\sigma_{m}$, and V are the unit normal vector, current density, conductivity of the membrane, and electric potential, respectively. The subscripts ‘int’ and ‘ext’ denote intracellularly and extracellularly, respectively.

The electric insulation boundary condition

(3) n·J=0

was implemented on the outer boundaries of the computational domain.

A constant voltage of 1 V was applied to one electrode using the Dirichlet boundary condition [100], and the other electrode was assigned to the ground. Since the CI operates in the electrical low-frequency regime and the region of interest is sufficiently small, we can safely assume that all cochlear tissues are purely resistive. Consequently, the quasi-static approximation [101,102] was used and the electric-field distribution in RC was derived by solving the constitutive equations:

(4) $$\begin{array}{c} J=\sigma E\\ E=-\nabla V\\ \nabla \cdot \left(\nabla V\right)=0 \end{array}\}.$$

Although the irregular geometry of neural tissues in RC was bypassed using the afore-described image method (Section 4.2), the heterogeneous distribution of conductivity values might still lead to large interpolation errors at the tissue interfaces. By controlling the mesh size with the Im(x,y) function, a virtual boundary was created to minimize possible interpolation issues. A mesh convergence study was performed to choose the best mesh that minimizes interpolation errors (details are provided in Appendix A). The final mesh consists of 226,306 triangular elements. The problem was solved for 457,882 degrees of freedom using a multifrontal massively parallel sparse direct solver (MUMPS) [103]. The total time taken to solve all parameters was 3 min and 24 s on the Windows server workstation with 64-Bit Intel^®^ Xenon^®^ CPU with 3.40 GHz (two processors) with 256 GB RAM.

## Figures and Tables

**Table 1:** Dimensions of the simulated subdomains.

Subdomain	Dimensions
Temporal bone	0.5 cm × 0.5 cm
Modiolus bone	100 µm × 2 mm
Electrode	0.3 mm (diameter)
Scala tympani	1 mm × 1 mm
Rosenthal’s canal	250 µm (diameter)
Spiral ganglion neurons	20–30 µm (diameter)

**Table 2:** Electric conductivity values of cochlear tissues used in the simulation

Subdomain	Electric Conductivity (S/m)
Temporal bone	0.016
Modiolus bone	0.0334
Electrode	9 × 10^6^
Tympanic medium	1.43
GroupA tissues	4 × 10^−9^
Intracellular medium	0.31
Extracellular medium	1.2
GroupB tissues	3.45 × 10^−6^

**Figure A1:**
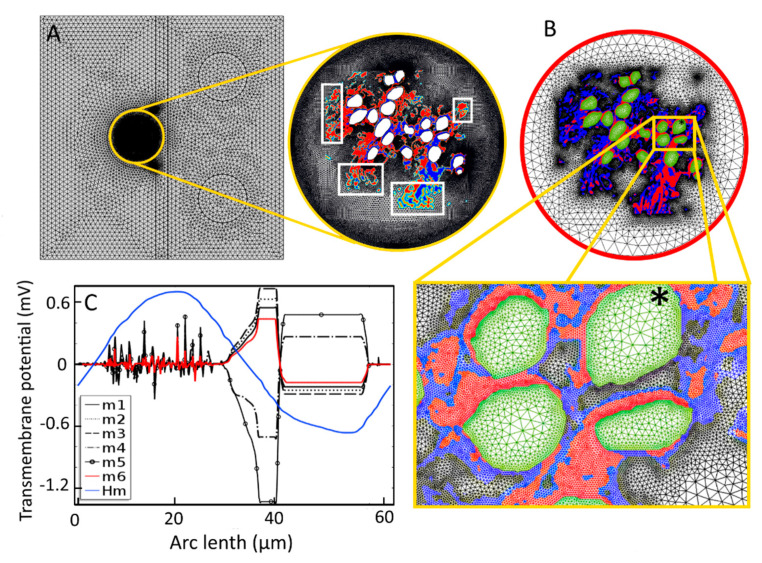
Meshing for the finite-element analysis. (**A**) Automatically generated triangular mesh by COMSOL Multiphysics^®^5.3a resulted in 251,030 elements in the whole computational domain. Conductivity distributions for GroupA and GroupB tissues are shown in blue and red, respectively. Intermediate colors shown in white boxes in the zoomed Rosenthal’s canal indicate the interpolation errors of electrical conductivity. (**B**) Final mesh (m6), accepted for the simulation and generated by Equation (A1), resulted in 226,306 mesh elements. The interpolation errors are diminished at the interfaces due to the virtual boundaries created by the adaptive mesh (zoomed picture). Here, conductivity distributions of GroupA and GroupB tissues are shown in red and blue, respectively. Green indicates the conductivity of intracellular medium. Black star shows C5 where the transmembrane potential is measured. (**C**) Transmembrane potential measured on C5 for seven mesh cases (m1 through to Hm) created by varying mesh parameters. The curve shown for Hm is for a homogeneous extracellular medium (shown in blue), and m6 (shown in red) is the result for the final accepted mesh.

## Abbreviations

AN	Auditory Nerve
AP	Action Potential
CI	Cochlear Implant
SGN	Spiral Ganglion Neuron
RC	Rosenthal’s Canal
$V_{m}$	Transmembrane Potential

## Appendix A. Mesh Convergence Study

A mesh convergence study was performed to minimize numerical error caused by the irregular forms of the tissues in Rosenthal’s canal (RC). Efficient, automatic, and adaptive meshing options are available in COMSOL Multiphysics^®^5.3a. However, the present two-dimensional model of the region of interest in RC poses a unique challenge when using automatic meshing options. Normally, meshing the computational domain should be straightforward, because the irregular and heterogeneously distributed edges of the tissues are not present in the model. However, even the finest mesh generated by automatic meshing options resulted in large interpolation errors when the conductivities of GroupA and GroupB tissues were mapped into the computational domain (Figure A1). To address this issue, we used a customized, local adaptive meshing method, which was controlled by the image data; the algorithm is defined as:

(A1) Mesh parameters=Finer on GroupA tissues,0.15<Im(x,y)≤0.3Fine on GroupB tissues,  0.05<Im(x,y)≤(0.15)Normal on ext. medium,      Im(x,y)≤(0.05).

By taking the mesh parameters shown in Table A1 as mesh case 6 (m6), five more mesh cases (m5–m1) were studied in which the mesh size was increased by a factor of two for GroupA tissues and by a factor of four for GroupB tissues for each consecutive case. The transmembrane potential, V_m_, of C5 was estimated for each mesh case. Due to the coarser sizes of the meshes, the m1–m5 cases showed large deviations in V_m_. A further reduction of the mesh size below that for m6 did not yield a change in V_m_. Hence, the final mesh (m6) was selected based on the study results shown in Figure A1. The final mesh parameters are given in Table A1, where Max, Min, GR, CF, and Res represent, respectively, the maximum element size, minimum element size, element growth rate, curvature factor, and resolution of the narrow regions. The accepted numerical error for calculating V_m_ was 10^−6^ mV.

**Table A1:** Mesh parameters used in the convergence study.

Mesh Type	Max	Min	GR	CF	Res
Finer	0.1 mm	0.6 µm	1.3	0.3	1
Fine	1 mm	6 µm	1.3	0.3	1
Normal	20 mm	4 µm	1.4	0.4	1

## Appendix B. Activating Function

A maximum of the activating function, defined as the second derivative of the extracellular potential, can provide an indication of the most likely AP initiation site [71]. Figure A2 shows the activating function for the 19 SGNs and six tissue samples used in the study. The sites of the maxima change with the tissue density, in what appears to be a random fashion, suggesting an unpredictable dependence of the AP initiation sites on tissue density. The AP initiation sites predicted by the maxima of the activation function and the transmembrane potential are different for different tissue samples (Figure A3).
